# Alignment of substance use community benefit prioritization and service lines in US hospitals: a cross-sectional study

**DOI:** 10.1186/s13722-024-00442-0

**Published:** 2024-02-13

**Authors:** Cory E. Cronin, Luke Kubacki, Lauren Donovan, Neeraj Puro, Dakota Lavinder, Kristin Schuller, Berkeley Franz

**Affiliations:** 1 College of Health Sciences and Professions, Ohio University, Athens, OH 45701 USA; 2 Appalachian Institute to Advance Health Equity Science, Ohio University, Athens, OH 45701 USA; 3 Heritage College of Osteopathic Medicine, Ohio University, Athens, OH 45701 USA; 4 College of Business, Florida Atlantic University, 777 Glades Rd, Boca Raton, FL 33431 USA; 5 College of Arts and Sciences, Ohio University, Athens, OH 45701 USA; 6 College of Health Professions, Towson University, 8000 York Rd, Towson, MD 21252 USA

**Keywords:** Hospitals, Community benefit, Substance use

## Abstract

**Background:**

Non-profit hospitals in the U.S. are required by the 2010 Patient Protection and Affordable Care Act (ACA) to conduct a community health needs assessment (CHNA) every three years and to formulate an implementation strategy in response to those needs. Hospitals often identify substance use as a need relevant to their communities in their CHNAs and then must determine whether to create strategies to address such a need within their implementation strategies. The aim of this study is to assess the relationship between a hospital’s prioritization of substance use within its community benefit documents and its substance use service offerings, while considering other hospital and community characteristics.

**Methods:**

This study of a national sample of U.S. hospitals utilizes data collected from publicly available CHNAs and implementation strategies produced by hospitals from 2018 to 2021. This cross-sectional study employs descriptive statistics and multivariable analysis to assess relationships between prioritization of substance use on hospital implementation strategies and the services offered by hospitals, with consideration of community and hospital characteristics. Hospital CHNA and strategy documents were collected and then coded to identify whether the substance use needs were prioritized by the hospital. The collected data were incorporated into a data set with secondary data sourced from the 2021 AHA Annual Survey.

**Results:**

Multivariable analysis found a significant and positive relationship between the prioritization of substance use as a community need on a hospital’s implementation strategy and the number of the services included in this analysis offered by the hospital. Significant and positive relationships were also identified for five service categories and for hospital size.

**Conclusions:**

The availability of service offerings is related both to a hospital’s prioritization of substance use and to its size, indicating that these factors are likely inter-related regarding a hospital’s sense of its ability to address substance use as a community need. Policymakers should consider why a hospital may not prioritize a need that is prevalent within their community; e.g., whether the organization believes it lacks resources to take such steps. This study also highlights the value of the assessment and implementation strategy process as a way for hospitals to engage with community needs.

## Introduction

While the global coronavirus pandemic monopolized much of the world’s healthcare-related attention since 2020, drug-related overdose deaths have continued to rise largely fueled by the ongoing opioid epidemic. Opioid-related overdose deaths have increased by over eight times since the turn of the millennium; data from the Centers for Disease Control and Prevention put the number of deaths by opioid overdose at nearly 69,000 for 2020 [1]. That same year, American life expectancy fell by almost two years. While this drop was propelled by the death toll from the new virus, it continued a recent downward trend fueled by drug overdose deaths [2]. Nationwide, substance use disorders (SUDs) cost more than $500 billion per year [3]. As the clinical and public health interventions focused on Covid-19 are reabsorbed into our health systems’ routine operations, renewed attention should be focused on the multidimensional and cross-sector efforts to develop and scale-up evidence-based prevention and treatment strategies for SUDs.

While this issue is national in scope, many meaningful interventions are occurring on the local level, where hospitals are uniquely poised to address the impacts of untreated substance use disorders in their surrounding communities. Hospitals are on the frontlines of treatment because they treat acute overdoses in the emergency department (ED) and treated infections and injuries secondary to substance use [4, 5]. This has created a need for hospitals to effectively screen, initiate evidence-based treatment, and transition patients to care in community-based settings [6].

A variety of formal service approaches have been utilized. For example, harm-reduction programs, such as syringe service programs and naloxone distribution, housed within EDs have shown to increase—in some studies, quite significantly—patients’ pursuit of further care, both clinical and behavioral [7]. Some hospitals have developed interdisciplinary addiction consult services, which have shown promise in reducing readmissions [8] and increasing trust in the healthcare system, [9] even in a telehealth context utilized by many hospitals during the Covid-19 pandemic [10]. But even without personnel devoted specifically to addiction medicine, the initiation of medications for opioid use disorder (MOUD) within the hospital has demonstrated improved outcomes compared with other interventions, such as referral to outside providers [11].

Hospitals are also addressing SUDs beyond formal service provision, which has received less attention in the literature. Substance use is associated with socioeconomic factors such as unemployment, work-related injuries, and chronic stress [12, 13]. Some of hospitals' impact comes through their ambient role as “anchor institutions” that influence their local economic, professional, and social environments, which can in turn moderate some of the social determinants of substance use [14, 15]. Hospitals also adopt important targeted preventive initiatives aimed at the public health of their local environment, [16] and they are further incentivized to do so via federal tax structures and state policy [17, 18]. But whether considering services offered within the hospital or initiatives within the broader community, the question to consider is whether it is need alone that contributes to a hospital’s decision to address SUDs, or whether other structural factors come into play.

Nearly 60% of hospitals in the US are non-profit entities and, as such, are required by the 2010 Patient Protection and Affordable Care Act (ACA) to conduct a community health needs assessment (CHNA) every three years and to formulate an implementation strategy (also referred to as strategy) in response to those needs [19, 20]. This process requires hospitals to engage with community stakeholders, particularly those with health expertise, to assess the needs of its community and to make its findings public, but is otherwise largely unstandardized [21] Hospital CHNAs often identify OUD, or substance use disorders more generally, as needs relevant to their communities [22, 23], and then must make the determination as to whether to create strategies to address such a need within their strategy.

Using these public hospital administrative documents as a starting point, the aim of this study was to assess the relationship between a hospital’s investment to address substance use within their community benefit documents and their formal offerings of substance use services in the clinical setting, while also taking other hospital and community characteristics into consideration. Doing so will provide insight into how hospitals make decisions in response to needs identified in the CHNA process, and what factors shape these decisions. In doing so, we utilized a novel dataset constructed from a national sample of hospital CHNA and implementation strategy documents, paired with hospital administrative data, which provides the first national comparison of hospital service lines to address SUDs and their community benefit programs related to substance use.

## Methods

### Sample

The sample for this study consists of a stratified, random sample of 20% of nonprofit hospitals in each state, rounded up to the nearest hospital (N = 601). We relied on a sample, rather than the full hospital population, due to the intensive work of coding CHNA and strategy documents.

We downloaded the publicly available CHNA and strategy from each hospital’s website. If these documents could not be located, we contacted the hospital by phone and/or email to request a copy. Hospitals that did not respond were removed from the sample. We utilized a coding strategy that was first used by the research team to analyze CHNAs and implementation strategies from an earlier wave of data for a previous study [22, 24]. This involved coding the top five health needs identified in the hospital CHNA, and the top five health needs addressed in the corresponding strategy. We also coded dichotomous variables for whether substance use was addressed in the strategy.

Because a team of coders were involved with this labor-intensive task, we measured intercoder reliability after a period of structured training and supervision. CHNAs and strategies typically follow a structured approach to reporting identified needs and related program implementation. We reached 100% reliability during a process of test coding. In situations where reports did not conform to the typical structure, we met as a team on a bi-weekly basis to collaboratively code all reports that were flagged. We also compared our sample to the national population of hospitals, using t-tests and chi-square tests, to assess consistency of characteristics such as bed size, system membership, teaching status, and rural location. We found our sample to have a slightly higher average bed size (mean of 236 beds, compared to 178 in the AHA population), but the sample appeared to be representative otherwise.

The dataset was created by merging CHNA data with data from the 2021 American Hospital Association Annual Survey, [25] County Health Rankings, [26] and Area Resource File, [27] in order to incorporate organization and community variables for each hospital within the sample. Hospitals that did not have publicly available CHNA or implementation strategies were removed from this analysis, as were hospitals that did not respond to the substance use services section of the AHA Annual Survey. Hospitals that do not provide this information tend to be systematically smaller and less-resourced than those that do [28, 29].

The final analytic sample was 354 hospitals, after removing those with missing data after the merging of datasets and those that did not meet inclusion criteria (e.g., children’s hospitals). Because our analysis relies entirely on secondary and organizational data and does not utilize any identifiable private information, our institutional review board indicated that our study did not require human subjects review.

### Measures

The dependent variables for this study are whether hospitals offer certain SUD-related services. We analyzed each service category individually, and also the categories collectively as a count variable (0–6; see Table 1 for description of the six service categories). The variables medications for opioid use disorder (MOUD), substance use consulting, and substance use screening were constructed by taking the setting (primary care, ER, inpatient, and extended care) or treatment specific (OUD and SUD) variables, and then creating dichotomous variables of the service, regardless of setting (Yes/No), before being calculated into the count. The main independent variable is whether the hospital includes substance use disorders (SUD) in its implementation strategy (0 = no, 1 = yes). Additionally, we have included hospital characteristics (bed size, teaching status, and system membership) and county characteristics (community health indicator composite score of 1 to 4, uninsured rate, if the hospital is in a rural designated county, and the county overdose mortality rate), as well as controlling for region, in order to understand the organizational and environmental context in which the hospital is operating.

**Table 1:** Key measures and description

Measure	Description
Substance use strategy	Substance use appears in the top needs listed on the hospital document
Medications for opioid use disorder (MOUD)	Medications for opioid use disorder for substance use and/or opioid use disorder. Medications for opioid use disorder (MOUD) is the use of medications, in combination with counseling and behavioral therapies, to provide a "whole-patient" approach to the treatment of substance use disorders. Medications used in MOUD are approved by the Food and Drug Administration (FDA) and MOUD programs are clinically driven and tailored to meet each patient's needs
Substance use consultation	Addiction/substance use disorder consultation and liaison services in the settings of ER, inpatient care, primary care, and/or extended care. Consultation-liaison psychiatrists, medical physicians, or advance practice providers (APPs) work to help people suffering from a combination of mental and physical illness by consulting with them and liaising with other members of their care team
Substance use screening	Substance use disorder screening in the settings of ER, inpatient care, primary care, and/or extended care
SUD inpatient services	Provides diagnosis and therapeutic services to patients with alcoholism or other drug dependencies. Includes care for inpatient/residential treatment for patients whose course of treatment involves more intensive care than provided in an outpatient setting or where patient requires supervised withdrawal
SUD outpatient services	Organized hospital services that provide medical care and/or rehabilitative treatment services to outpatients for whom the primary diagnosis is alcoholism or other chemical dependency
Telehealth addiction services	Telepsychiatry can involve a range of services including psychiatric evaluations, therapy, patient education, and medication management

### Analytic plan

We employed descriptive statistics to establish frequencies, percentages or medians, and range, where appropriate, for each variable. All frequency and percentage variables were coded as 0/1, and all median and range variables were continuous variables. The analysis also utilized chi-square tests to assess significant associations between individual service offerings and the presence of SUD in a strategy. Additionally, a Poisson regression model was used to assess the relationship between the count of service offerings and the presence of SUD in a strategy, while controlling for hospital and community characteristics.

## Results

Our findings indicate that the majority of hospitals included in the sample (77%) have programming in SUD as part of their implementation strategy. An even higher number of hospitals (92%) offered at least one formal medical service to treat SUD (Table 2).

**Table 2:** Descriptive statistics; frequencies, means and range (N = 354)

	Frequency	Percent
Substance use in implementation strategy (IS)	273	77.12%
SUD Hospital Services		
Screening	302	85.31%
Consultation	263	74.29%
Inpatient	44	12.43%
Outpatient	85	24.01%
MOUD	96	27.12%
Telehealth	119	33.62%
Hospital bed size		
Fewer than 50	88	24.86%
50–199	114	32.20%
200–399	81	22.88%
400 or more	71	20.06%
Major teaching status	40	11.30%
System member hospital	271	76.55%
Community Health Indicator		
1 (Best)	139	39.27%
2	83	23.45%
3	74	20.90%
4 (Worst)	58	16.38%
County rural	48	13.56%
Region		
Northeast	77	21.75%
Midwest	117	33.05%
South	88	24.86%
West	72	20.34%

	Mean	Range
Count of services	2.57	(0–6)
Percent of county population uninsured	9.89%	(3–25.1%)
County overdose rate	15.71%	3.05–30

**Fig. 1:**
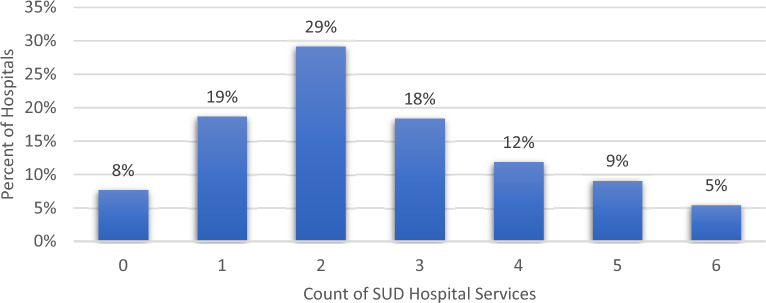
Percent of hospitals by count of SUD hospital services

The mean number of service categories offered by hospitals is 2.57, with two-thirds of the hospitals in the sample offering between one and three of the categories analyzed. Just under 8% reported no availability of the specified services, while just over 5% reported offering all six (see Fig. 1).

**Fig. 2:**
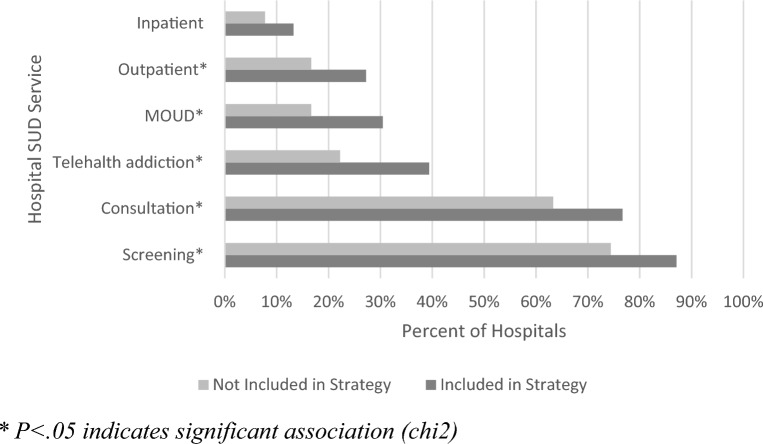
Service offering prevalence in relation to SUD presence in Implementation Strategies.*P < 0.05 indicates significant association (chi2)

When considering the service categories individually, each of them was positively associated with SUD being included in the implementation strategy, and all but inpatient services were statistically significant relationships (see Fig. 2).

Poisson regression analysis showed a significant and positive relationship between the SUD prioritization in a hospital strategy and count of service offerings, indicating that hospitals that prioritize SUD on their community benefit documents offer more services relevant to SUD. Other variables that show significant relationships to the count of services are hospital size, with larger hospitals offering more services; and region, with hospitals in the Northeast offering a greater number of services than the reference region of the South (Table 3).

**Table 3:** Multivariable Poisson regression; association of hospital and community characteristics with hospital count of offered SUD services N = 354

	B	(SE)	P	[95% conf. interval]	
SUD in hospital strategy	0.19	(0.09)	0.040	0.01 to 0.36	
Hospital size					
Fewer than 50 beds	Ref.				
50–199 beds	0.23	(0.11)	0.028	0.02 to 0.44	
200–399 beds	0.45	(0.11)	0.000	0.23 to 0.67	
More than 400 beds	0.73	(0.12)	0.000	0.49 to 0.97	
Major teaching hospital	− 0.08	(0.11)	0.473	− 0.30 to 0.14	
System member hospital	− 0.02	(0.08)	0.772	− 0.19 to 0.14	
Community Health Indicator					
1 (best)	Ref.				
2	0.01	(0.09)	0.971	− 0.18 to 0.18	
3	0.07	(0.10)	0.445	− 0.12 to 0.26	
4 (worst)	0.07	(0.10)	0.517	− 0.14 to 0.27	
Percent of county population uninsured	− 0.01	(0.01)	0.274	− 0.03 to 0.01	
County classified rural	− 0.12	(0.12)	0.323	− 0.37 to 0.12	
County overdose rate	0.01	(0.01)	0.206	− 0.01 to 0.02	
Region					
South	Ref.				
Midwest	0.22	(0.12)	0.061	− 0.01 to 0.45	
Northeast	0.40	(0.13)	0.002	0.15 to 0.65	
West	0.20	(0.12)	0.095	− 0.03 to 0.43	

Ref = reference group

## Discussion

The decisions hospitals make regarding service offerings and community benefit programs are often complex and multi-faceted. The Affordable Care Act introduced new requirements for hospitals to conduct CHNAs and develop implementation strategies, in part, to ensure that hospitals are developing community health programs that are in line with local needs, rather than organizational priorities. Our findings suggest that a hospital’s service offerings are significantly associated with whether they invested in addressing SUDs in prior years. Because our data did not allow us to consider whether the service pre-dated the strategy or not, we cannot conclude whether or not the service is a result of the committing to address substance use in their community, though that is certainly possible. However, we can recognize a clear relationship between the willingness to engage with a need at a community level and the ability to provide services within the clinical setting. Beyond the number of services offered, it is notable that the only service without statistical significance is the one perhaps considered to be the most traditional (in-patient care) and that the relationship to hospital investments in the community is stronger amongst those services that could be viewed as taking a more preventive approach to SUDs.

The findings of this study provide possible context to the decision-making process within hospitals, by indicating that hospitals may consider formal service offerings when addressing substance use in the community. In other words, hospitals may consider their formal services offerings as a foundation to build upon when publicly committing to address SUDs as part of their community benefit requirements. On the other hand, limitations in resources may both limit hospital service availability and community benefit investments. Small hospitals, in particular, may not have the economic resources or staffing to broaden their scope, no matter how high the need in the community may be [30]. Such findings provide support for ideas such as the “liability of smallness” [31]. It is plausible that addressing SUD within the broader community would help to control costs in the long run, given the potential to prevent overdoses and costly infections, but developing a community benefit strategy to address substance use may require considerable start-up resources and personnel. Yet, clinical services may not be the only way to address substance use, and there may be hospitals that recognize the need, and are willing to engage in it through partnerships with other local stakeholders [32, 33]. Such efforts also have the potential to create effective change, and hospitals that feel limited in their service offerings could consider such alternative options.

If hospitals lack the direct resources to create SUD interventions in the community, there is potential for state and local policymakers to direct funds to address such gaps. Many patients with SUD do interact with the medical system for a separate condition or injury, but do not have their primary SUD addressed [5, 34]. To effectively address the SUD epidemic, additional resources must be deployed to educate, train, and support health care organizations to undertake both preventive programming and formal service provision.

### Limitations

The primary limitation of this study is that it relies on the self-reported nature of implementation strategies. By gathering data from these reports, we are relying on the expressed intentions of hospitals, without verification that hospitals did adhere to their reported strategies. It is possible that some expressed strategies never came to fruition, although hospitals are required to provide an update on each strategy in their next round of reporting. It is also possible that hospitals did not report SUD as a priority at the time of the strategy, but later sought to address the issue in some way. A final limitation is the use of cross-sectional secondary data with a primary dichotomous outcome, with yes/no responses for key variables, which does not allow us to assess the degree of implementation or assess causality between formal service lines and community benefit investments.

## Conclusion

Hospital CHNAs and implementation strategies provide extensive information about the needs hospitals identify within their communities, and the steps they intend to take to address these health needs. Analysis of these documents can also help researchers and policymakers understand hospital decision-making and the complex range of factors associated with such decisions; identify where gaps between those needs and actions exist; and begin to understand what the implications are for improving community health. Organizations may view themselves equipped to address community health needs when they believe they have the ability and appropriate resources to implement effective interventions. Organizations that recognize limitations in their resources may feel constrained both in their ability to offer key services and in their ability to stretch into new areas of prevention, even when high needs are shown. With this understanding, policymakers can consider new actions to support organizations that have limited resources but serve high-need areas where new preventive efforts are critical.

## Data Availability

The dataset used in this study include data from publicly available hospital reports and data from the American Hospital Association. Because the American Hospital Association data were used under license for the current study, and the full dataset is not publicly available.

## Abbreviations

CHNA: Community Health Needs Assessment
MOUD: Medication for opioid use disorder
ACA: Patient Protection and Affordable Care Act
OUD: Opioid use disorder
ED: Emergency department
AHA: American Hospital Association
SUD: Substance use disorder
